# Extracellular matrix metalloproteinase inducer as a biomarker for oral cancer

**DOI:** 10.6026/97320630019433

**Published:** 2023-04-30

**Authors:** Chanchal Katariya, Arvina Rajasekar

**Affiliations:** 1 Department of Periodontics, Saveetha Dental College and Hospitals, SIMATS, CHENNAI-77

## Views:

Tumor cell invasion and metastasis are mediated by a variety of biological processes, including Cell-surface adhesion molecules are changed, the cytoskeleton is altered, and the extracellular matrix is penetrated (ECM). Malignant cells travel through the interstitial matrix and the basement membrane, two significant groups of extracellular matrixes, when they invade host tissues. Two different changes to the composition of these matrices are necessary for tumour cells to successfully penetrate the ECM: removal of extracellular matrix barriers that already exist and the creation of a new extracellular atmosphere that is conducive to tumour cell migration and proliferation. [1] A dynamic system that is partially powered by the production of matrix metalloproteinases controls the ECM's composition (MMPs). Dissolution of pre-existing matrix molecules and the renaissance of transitional matrices leads to the remodelling of the ECM. Destruction of pre-existing matrix molecules are usually regulated by a number of different molecules and proteins, one of them is MMPs. The greater MMP levels found in cancer specimens must be produced by tumor-associated fibroblasts, which are generally found to have moderate MMP levels. [2] Solvents secreted by tumour cells can induce fibroblasts in culture to produce large amounts of collagenase. [3] A glycoprotein was discovered by Biswas and Nugent as the likely cause of this effect. These days, this glycoprotein is identified as EMMPRIN (extracellular matrix metalloproteinase inducer; previously named tumour cell-derived collagenase stimulatory factor, or TCSF). EMMPRIN is a transmembrane protein that weighs 45-55 kDa and is heavily glycosylated. It belongs to the superfamily of immunoglobulins. [4] Tumor cells have the ability to secrete EMMPRIN in a soluble form as well. Several malignancies have been linked to the stimulation of fibroblast MMP production that is seen in vivo, may be at least in part caused by EMMPRIN from tumour cells, whether it is in soluble or membrane form. EMMPRIN takes role in metastasis and encroachment by promoting adjacent fibroblasts to release more interstitial collagenase and gelatinase, which helps tumour cells move through the surrounding matrix. [5]

It participates in a number of carcinogenesis-related processes that start and advance malignancy and is significantly expressed in a variety of malignant neoplasms. [6] Unrelated to the model study, a literature meta-analysis found a significant link between Poor tumour outcomes, such as overall survival, disease-specific survival, progression-free survival, metastasis-free survival, or recurrence-free survival, are associated with EMMPRIN overexpression. Additionally, the overexpression of CD147/EMMPRIN suggested a higher risk of chemotherapeutic treatment resistance. [7] EMMPRIN activates a number of matrix metalloproteinases molecules, including membrane-type 1-MMP, MMP-1, MMP-3, and MMP-9, facilitating the proliferation, invasion, and migration of tumor cells. [8] EMMPRIN is associated with many proteins that are involved in different signalling pathways, including integrins, caveolin-1 (Cav-1), tenascin (TN)-C, urokinase-type plasminogen activator (uPA), matrix metalloproteinases, ErbB, MAPK cascade proteins, monocarboxylate transporters (MCT), and cyclophilins (Cyp)[9] EMMPRIN has the ability to increase angiogenesis in the tumour microenvironment by inducing vascular endothelial growth factors in tumour and stromal cells. [10] The urge for survival that causes metabolic alteration in tumour cells is a hallmark of carcinogenesis. EMMPRIN creates complexes on the membrane and regulates the expression and activity of monocarboxylate transporters-1 (MCT-1) and MCT-4 to transfer the lactic acid generated by anaerobic glycolysis. [11] It has been discovered that EMMPRIN facilitates the impulse of the phosphatidylinositol 3-hydroxy kinase and mitogen-activated protein kinase pathways, which are essential steps in the progression of cancer and primarily responsible for the chemoresistance of tumour cells. Collagen, laminin, and fibronectin are examples of extracellular matrix proteins to which adherence must be controlled; EMMPRIN also interacts with integrins 3 and 6. Additionally, it promotes cyclophilin A expression to speed up the development of cancer cells. [12] In oral premalignant cells as well as main and metastatic OSCC cell lines, increased expression of EMMPRIN has been found. [13]

All of the signalling pathways outlined above have been found to influence the etiology of oral cancer. EMMPRIN overexpression was shown to be more prevalent in moderately/poorly differentiated (G2/G3) cancers than in well-differentiated (G1) tumors, suggesting a correlation between EMMPRIN expression and histological grade. [14] It was discovered that a considerable increase in EMMPRIN expression compared to healthy oral mucosa and a robust expression in more than 90% of tumour cells from early-invasive OSCC and in situ carcinoma. [15] The investigators also discovered that the degree of dysplasia was inversely linked with the level of this marker's expression in oral leukoplakias, indicating that early oral cancer development is accompanied by the overexpression of EMMPRIN. and aids in the development of oral tumours. OSCC patients may therefore be at a significant risk for COVID-19 due to the elevated expression and availability of EMMPRIN. Additionally, COVID-19 may exhaust EMMPRIN in OSCC patients as a result of binding to the "S" receptor and disrupt associated carcinogenesis events, as shown above. The old theory, according to which a reduction in ACE-2 receptors in OSCC patients would cause this environment to be less susceptible to COVID-19, is at odds with the present theory. [16, 17, 18, 19, 20, 21, 22, 23, 24, 25, 26]

## References

[1] Bordador LC (2000). International Journal of Cancer..

[2] Mann EA (1995). Annals of Otology Rhinology and Laryngology..

[3] Biswas C, Toole BP (1987). Cell Membranes..

[4] Berditchevski F (1997). J Biol Chem..

[5] Guo H (1997). Journal of Biological Chemistry..

[6] Xin X (2016). Sci Rep.

[7] Bovenzi CD (2015). Biomed Res Int.

[8] Huang J (2014). International Journal of Gynecologic Cancer..

[9] Iacono KT (2007). Experimental and Molecular Pathology..

[10] Pinheiro C (2015). BMC Cancer.

[11] Li S (2014). Invest Ophthalmol Vis Sci.

[12] Huang C (2012). Histopathology.

[13] Vigneswaran N (2006). Exp Mol Pathol..

[14] Monteiro LS (2014). Biomed Res Int..

[15] Vigneswaran N (2006). Experimental and Molecular Pathology..

[16] Sarode SC (2020). Oral Oncology..

[17] Asha P (2022). Environ Res..

[18] Shanmugam R (2021). Energy Sources Part A..

[19] Johnson J (2020). Hypertens Res..

[20] Mathivadani V (2020). Acta Virol..

[21] Uma Maheswari TN (2020). Braz Oral Res..

[22] Ma Y, Karunakaran T (2019). Biotechnol Bioprocess Eng..

[23] Ponnanikajamideen M (2019). Can J Diabetes..

[24] Aurtherson PB (2021). Biomass Conversion and Biorefinery.

[25] Adhinarayanan R (2020). Energy Sources Part A..

[26] Thanikodi S (2020). Therm Eng.

